# Supplementary material for: Unveiling the Bioactive Potential of the Invasive Jellyfish Phyllorhiza punctata Through Integrative Transcriptomic and Proteomic Analyses
Source: Biomolecules. 2025 Aug 4;15(8):1121. doi: 10.3390/biom15081121 (PMC12383608; doi:10.3390/biom15081121)
Supplement: Supplementary file 1 [file biomolecules-15-01121-s001.zip › Suplementary Material/FileS3/PEPFOLD4/XP_065055256_10617/clusters.html]

 PEP-FOLD clusters


Download archive of all models  here   

|  |  |  |
| --- | --- | --- |
| # | file | sOPEP |
| # cluster 1 - Size: 57 |  |  |
|  | XP\_065055256\_10617-model1 | -20.274 |
|  | XP\_065055256\_10617-model1.1 | -20.1163 |
|  | XP\_065055256\_10617-model1.2 | -19.8708 |
|  | XP\_065055256\_10617-model1.3 | -19.8496 |
|  | XP\_065055256\_10617-model1.4 | -19.8099 |
|  | XP\_065055256\_10617-model1.5 | -19.7974 |
|  | XP\_065055256\_10617-model1.6 | -19.6568 |
|  | XP\_065055256\_10617-model1.7 | -19.421 |
|  | XP\_065055256\_10617-model1.8 | -19.4086 |
|  | XP\_065055256\_10617-model1.9 | -19.3827 |
|  | XP\_065055256\_10617-model1.10 | -19.3651 |
|  | XP\_065055256\_10617-model1.11 | -19.3406 |
|  | XP\_065055256\_10617-model1.12 | -19.3285 |
|  | XP\_065055256\_10617-model1.13 | -19.3096 |
|  | XP\_065055256\_10617-model1.14 | -19.3013 |
|  | XP\_065055256\_10617-model1.15 | -19.1362 |
|  | XP\_065055256\_10617-model1.16 | -19.0048 |
|  | XP\_065055256\_10617-model1.17 | -18.9905 |
|  | XP\_065055256\_10617-model1.18 | -18.9867 |
|  | XP\_065055256\_10617-model1.19 | -18.9566 |
|  | XP\_065055256\_10617-model1.20 | -18.9459 |
|  | XP\_065055256\_10617-model1.21 | -18.9398 |
|  | XP\_065055256\_10617-model1.22 | -18.9097 |
|  | XP\_065055256\_10617-model1.23 | -18.8985 |
|  | XP\_065055256\_10617-model1.24 | -18.8896 |
|  | XP\_065055256\_10617-model1.25 | -18.8509 |
|  | XP\_065055256\_10617-model1.26 | -18.8328 |
|  | XP\_065055256\_10617-model1.27 | -18.8296 |
|  | XP\_065055256\_10617-model1.28 | -18.8228 |
|  | XP\_065055256\_10617-model1.29 | -18.7977 |
|  | XP\_065055256\_10617-model1.30 | -18.787 |
|  | XP\_065055256\_10617-model1.31 | -18.7575 |
|  | XP\_065055256\_10617-model1.32 | -18.7249 |
|  | XP\_065055256\_10617-model1.33 | -18.7179 |
|  | XP\_065055256\_10617-model1.34 | -18.7133 |
|  | XP\_065055256\_10617-model1.35 | -18.7024 |
|  | XP\_065055256\_10617-model1.36 | -18.7017 |
|  | XP\_065055256\_10617-model1.37 | -18.6882 |
|  | XP\_065055256\_10617-model1.38 | -18.6878 |
|  | XP\_065055256\_10617-model1.39 | -18.6793 |
|  | XP\_065055256\_10617-model1.40 | -18.6766 |
|  | XP\_065055256\_10617-model1.41 | -18.6745 |
|  | XP\_065055256\_10617-model1.42 | -18.6555 |
|  | XP\_065055256\_10617-model1.43 | -18.6213 |
|  | XP\_065055256\_10617-model1.44 | -18.5669 |
|  | XP\_065055256\_10617-model1.45 | -18.5568 |
|  | XP\_065055256\_10617-model1.46 | -18.5298 |
|  | XP\_065055256\_10617-model1.47 | -18.5272 |
|  | XP\_065055256\_10617-model1.48 | -18.522 |
|  | XP\_065055256\_10617-model1.49 | -18.5098 |
|  | XP\_065055256\_10617-model1.50 | -18.4357 |
|  | XP\_065055256\_10617-model1.51 | -18.4318 |
|  | XP\_065055256\_10617-model1.52 | -18.2959 |
|  | XP\_065055256\_10617-model1.53 | -18.2201 |
|  | XP\_065055256\_10617-model1.54 | -18.1213 |
|  | XP\_065055256\_10617-model1.55 | -18.0984 |
|  | XP\_065055256\_10617-model1.56 | -18.005 |
| # cluster 2 - Size: 10 |  |  |
|  | XP\_065055256\_10617-model2 | -19.1841 |
|  | XP\_065055256\_10617-model2.1 | -19.1218 |
|  | XP\_065055256\_10617-model2.2 | -18.8905 |
|  | XP\_065055256\_10617-model2.3 | -18.8013 |
|  | XP\_065055256\_10617-model2.4 | -18.7903 |
|  | XP\_065055256\_10617-model2.5 | -18.7286 |
|  | XP\_065055256\_10617-model2.6 | -18.597 |
|  | XP\_065055256\_10617-model2.7 | -18.1542 |
|  | XP\_065055256\_10617-model2.8 | -18.0881 |
|  | XP\_065055256\_10617-model2.9 | -18.0686 |
| # cluster 3 - Size: 10 |  |  |
|  | XP\_065055256\_10617-model3 | -19.1415 |
|  | XP\_065055256\_10617-model3.1 | -18.8366 |
|  | XP\_065055256\_10617-model3.2 | -18.8117 |
|  | XP\_065055256\_10617-model3.3 | -18.7136 |
|  | XP\_065055256\_10617-model3.4 | -18.705 |
|  | XP\_065055256\_10617-model3.5 | -18.5957 |
|  | XP\_065055256\_10617-model3.6 | -18.5092 |
|  | XP\_065055256\_10617-model3.7 | -18.3771 |
|  | XP\_065055256\_10617-model3.8 | -18.218 |
|  | XP\_065055256\_10617-model3.9 | -18.1426 |
| # cluster 4 - Size: 15 |  |  |
|  | XP\_065055256\_10617-model4 | -19.1337 |
|  | XP\_065055256\_10617-model4.1 | -18.9662 |
|  | XP\_065055256\_10617-model4.2 | -18.9487 |
|  | XP\_065055256\_10617-model4.3 | -18.8682 |
|  | XP\_065055256\_10617-model4.4 | -18.7192 |
|  | XP\_065055256\_10617-model4.5 | -18.7013 |
|  | XP\_065055256\_10617-model4.6 | -18.6985 |
|  | XP\_065055256\_10617-model4.7 | -18.698 |
|  | XP\_065055256\_10617-model4.8 | -18.6655 |
|  | XP\_065055256\_10617-model4.9 | -18.5515 |
|  | XP\_065055256\_10617-model4.10 | -18.4384 |
|  | XP\_065055256\_10617-model4.11 | -18.432 |
|  | XP\_065055256\_10617-model4.12 | -18.2611 |
|  | XP\_065055256\_10617-model4.13 | -18.2428 |
|  | XP\_065055256\_10617-model4.14 | -18.2408 |
| # cluster 5 - Size: 4 |  |  |
|  | XP\_065055256\_10617-model5 | -19.0134 |
|  | XP\_065055256\_10617-model5.1 | -18.7984 |
|  | XP\_065055256\_10617-model5.2 | -18.657 |
|  | XP\_065055256\_10617-model5.3 | -18.5274 |
| # cluster 6 - Size: 4 |  |  |
|  | XP\_065055256\_10617-model6 | -18.5989 |
|  | XP\_065055256\_10617-model6.1 | -18.4544 |
|  | XP\_065055256\_10617-model6.2 | -18.3567 |
|  | XP\_065055256\_10617-model6.3 | -18.167 |
|  |  |  |
